# Supplementary material for: The MYB-like protein MylA contributes to conidiogenesis and conidial germination in Aspergillus nidulans
Source: Commun Biol. 2024 Jun 25;7:768. doi: 10.1038/s42003-024-05866-7 (PMC11199622; doi:10.1038/s42003-024-05866-7)
Supplement: Supplementary file 2 — Supplementary Information [file 42003_2024_5866_MOESM2_ESM.pdf]

Supplemental information

A MYB-like protein MylA contributes to conidiogenesis and conidial germination in *Aspergillus nidulans*

Ye-Eun Son[1], He-Jin Cho[1], and Hee-Soo Park[1,2*]

Corresponding Author:

Hee-Soo Park, phsoo97@knu.ac.kr

This PDF file includes:

Supplementary Figures 1 to 6

Supplementary Table 1

Supplementary References

Supplementary Figures

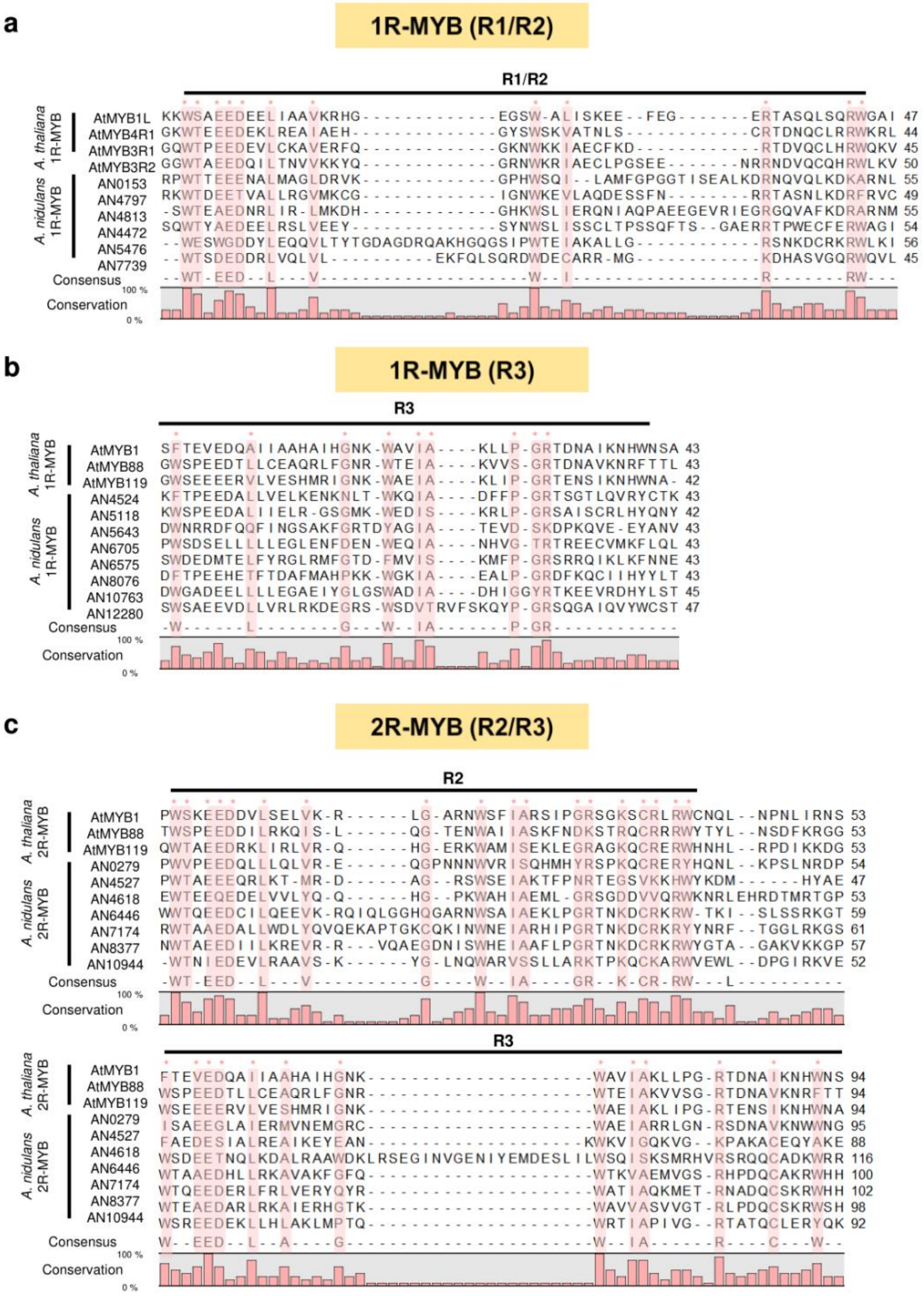

Supplementary Fig 1. Sequence alignment of MYB repeats in MYB-like proteins

a R1/R2 repeat domain alignment of 1R-MYB constructed using CLC Sequence Viewer 8. **b** R3 repeat domain alignment of 1R-MYB constructed using CLC Sequence Viewer 8. **c** R2 and R3 repeat domain alignment of 2R-MYB constructed using CLC Sequence Viewer 8. Representative MYB repeat sequences from *Arabidopsis thaliana* were aligned with the MYB repeat sequences of each gene from *A. nidulans*. Pink box and asterisks designate consensus amino acid sequences (conservation rate >60%).

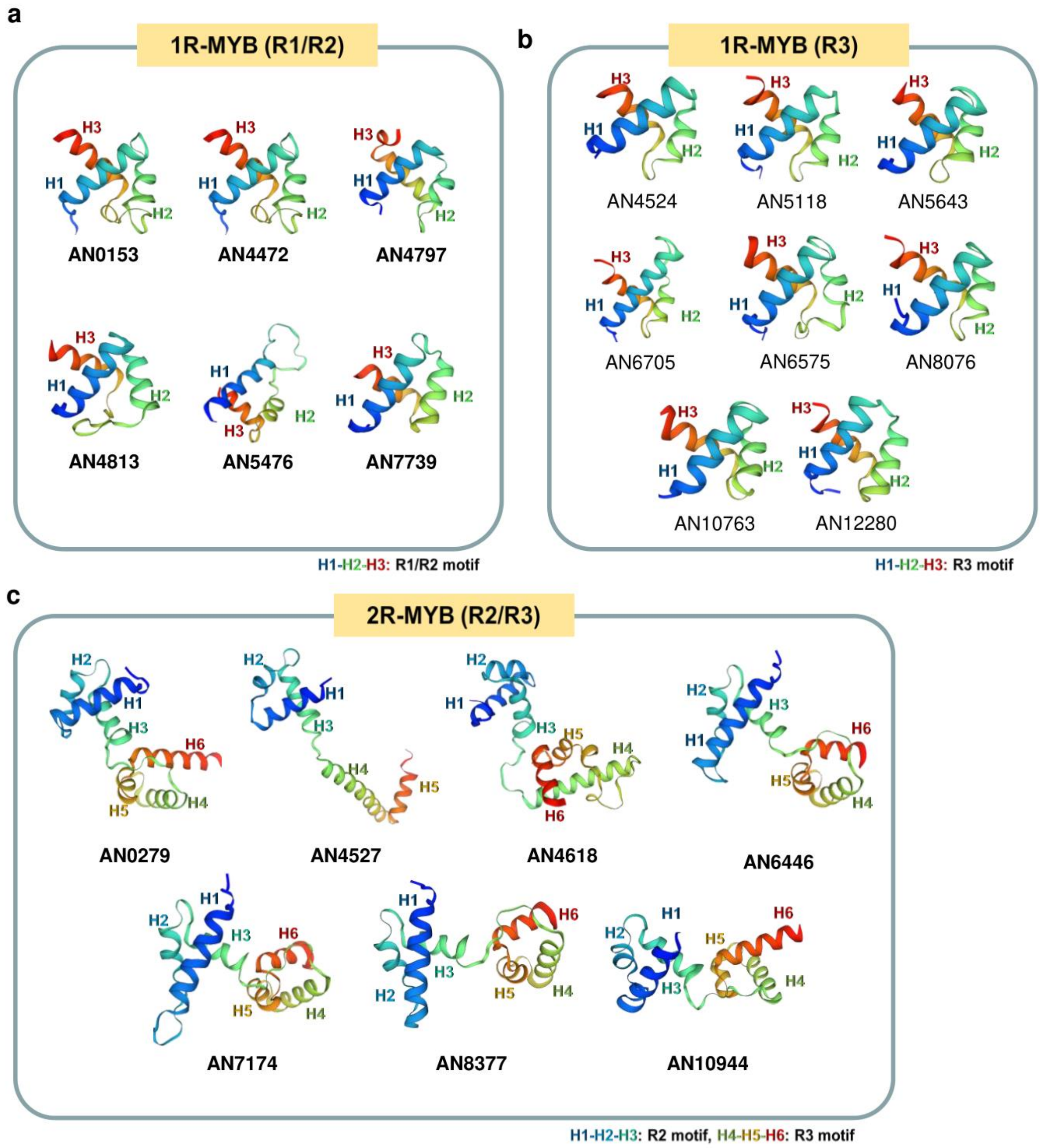

Supplementary Fig 2. Classification of 21 MYB-like proteins in *A. nidulans*

a Secondary structures of six 1R MYB-like proteins had R1/R2 repeat motif were predicted using SWISS-MODEL. The consensus sequence of R1/R2 was WT-(X_1)-EED-(X_2)-L-(X_3)-V…W-(X_2)-I…R-(X_7)-RW. **b** Secondary structures of eight 1R MYB-like proteins had R3 repeat motif were predicted using SWISS-MODEL. The consensus sequence of R3 was W-(X_6)-L-(X_8)-G-(X_2)-W-(X_2)-IA…PGR…F/Y/W. **c** Secondary structures of seven 2R MYB-like proteins in *A. nidulans*, which were

predicted using SWISS-MODEL. R2 and R3 conserved motifs were WT-(X_1)-EED-(X_2)-L-(X_3)-V…G…W-(X_2)-IA…GR-(X_{2-})K-(X_1)-CR-(X_1)-RW and W-(X_2)-EED-(X_2)-L-(X_2)-A-(X_4)–G…-W-(X_2)-IA…R-(X_4)-C-(X_3)-W, respectively.

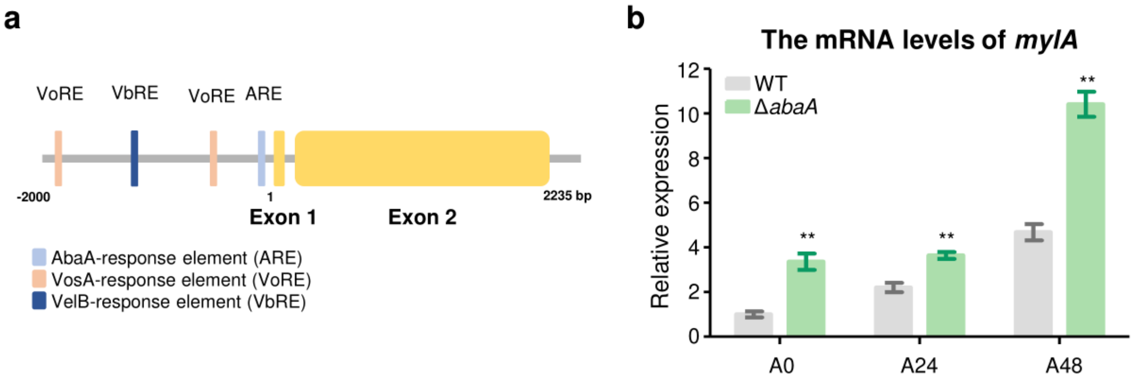

Supplementary Fig 3. The genetic relationship between MylA and AbaA.

a Schematic diagram showing the binding sites of AbaA, VosA, and VelB. The start codon is at "1" and the stop codon is at "2235". In scanning the *mylA* promoter region up to 2000 base pair, AbaA-response element (ARE, 5′-CATTCY-3′, Y= C or T) is existed in "-52". VosA-response element (VoRE, 5′-CCNNGG-3′, N= any base) is existed in "-1871" and "-602" and VelB-response element (VbRE, 5′-CCNTGG-3′, N= any base) is existed in "-1263". **b** Relative expression level of the *mylA* gene in WT and Δ*abaA* strains after post-asexual developmental induction. The mRNA expression was normalized to that of the endogenous control β-actin gene (**p < 0.01).

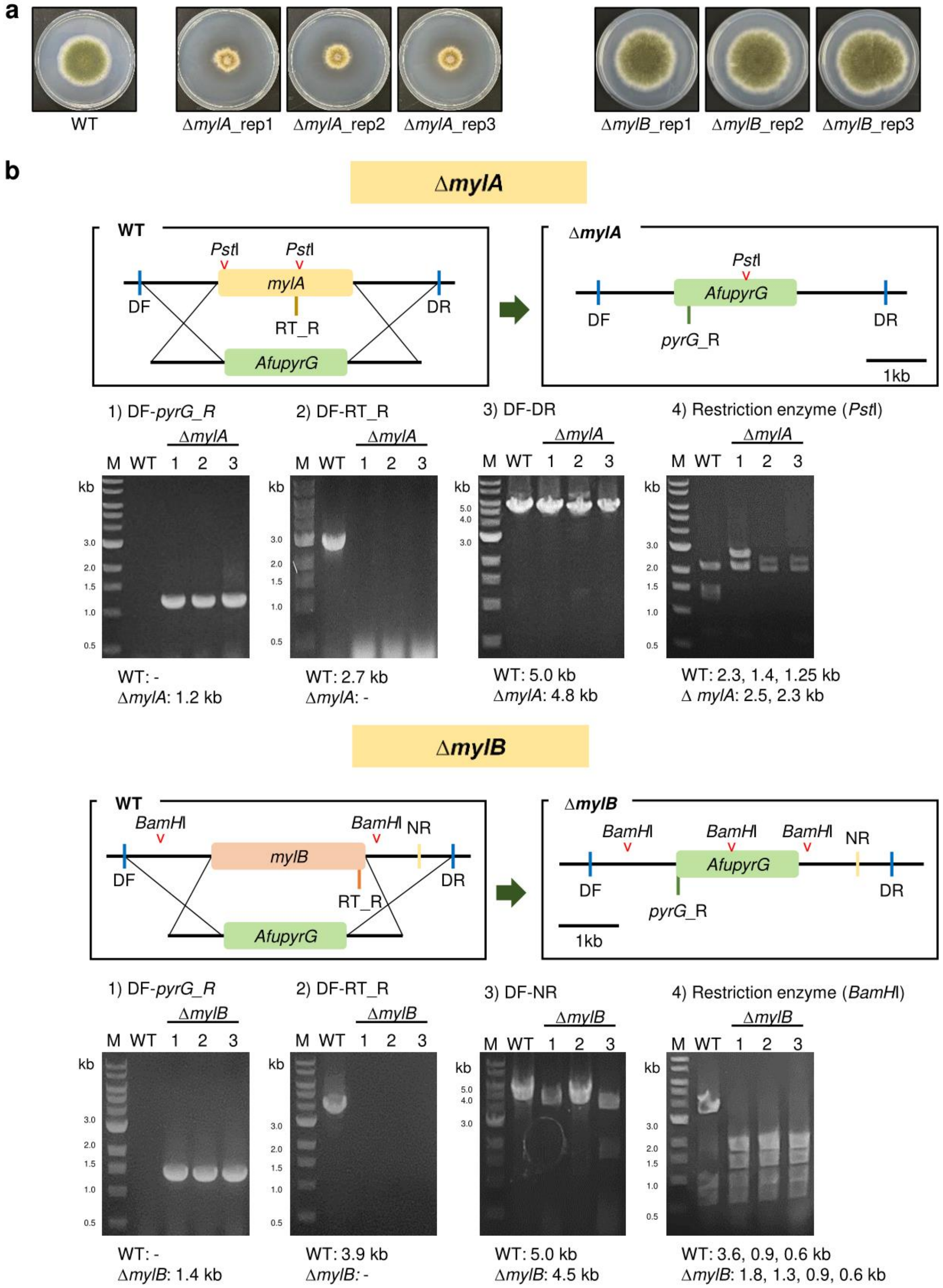

Supplementary Fig 4. Verification of the *mylA* and *mylB* deletion strains

a Colony morphology of WT, *mylA* or *mylB* deletion strains. At least three independent

mutant strains were generated. **b** Schematic illustration of strategy used to generate Δ*mylA* or Δ*mylB* mutant strains. Below pictures indicated PCR verification of Δ*mylA* or Δ*mylB* mutant strains.

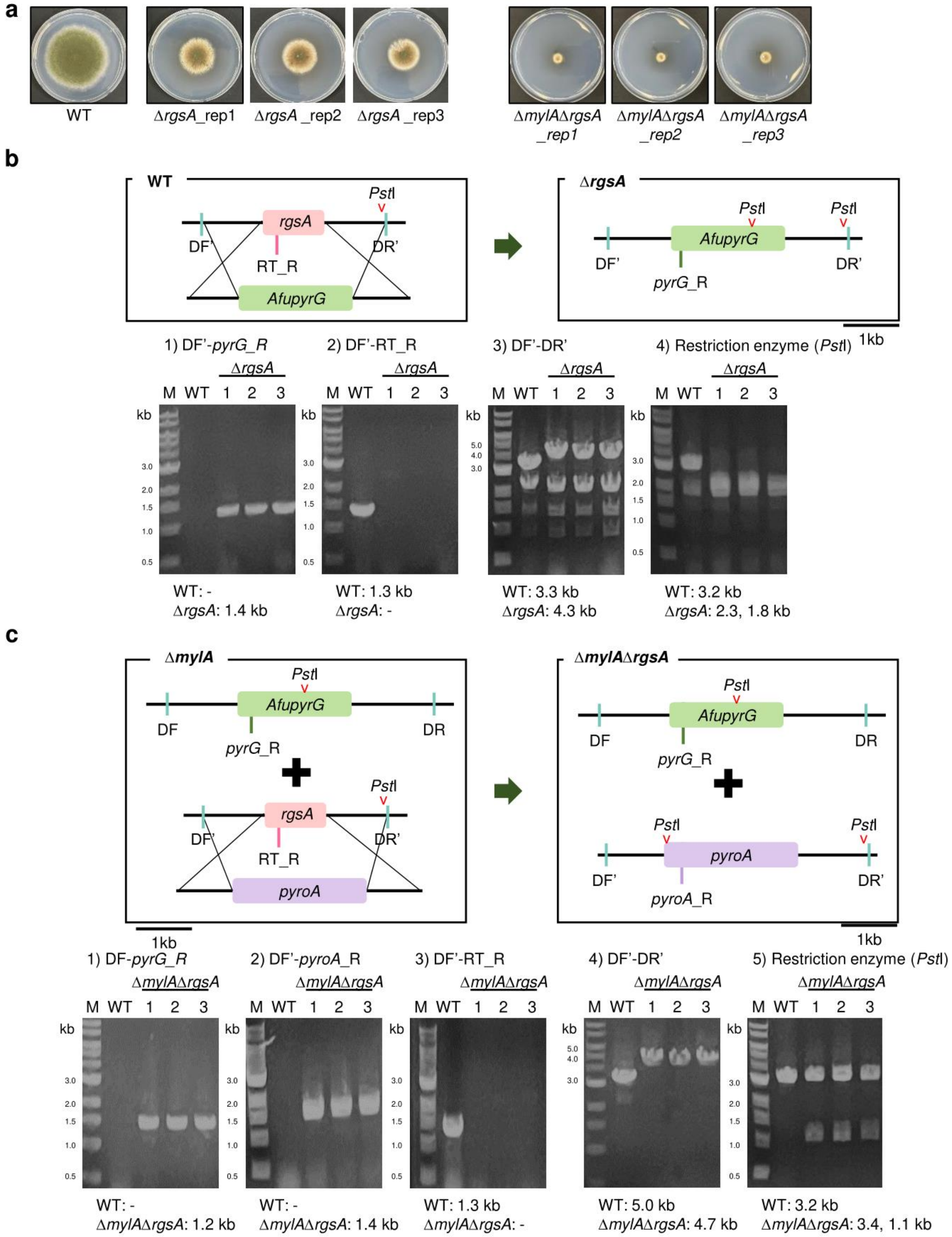

Supplementary Fig 5. Verification of the *rgsA* single deletion and the *mylArgsA* double deletion strains.

a Colony morphology of WT, *rgsA* single or *mylArgsA* double deletion strains. At least

three independent mutant strains were generated. **b** Schematic illustration of strategy used to generate Δ*rgsA* mutant strains. Below pictures indicated PCR verification of Δ*rgsA* mutant strains. **c** Schematic illustration of strategy used to generate Δ*mylArgsA* mutant strains. Below pictures indicated PCR verification of Δ*mylArgsA* mutant strains.

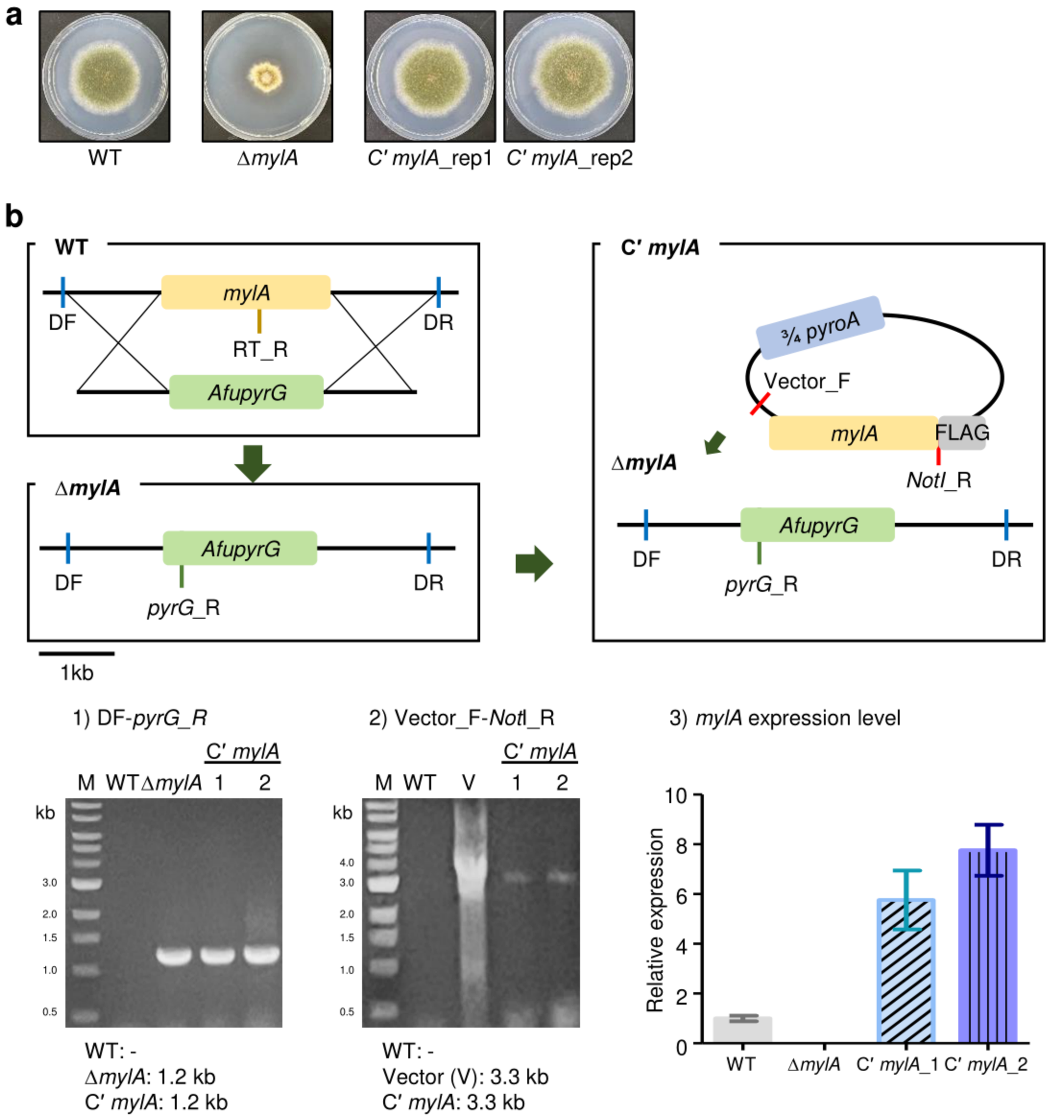

Supplementary Fig 6. Verification of the *mylA*-complementary strains

a Colony morphology of WT, Δ*mylA* or C′ *mylA* strains. At least two independent complementary strains were generated. **b** Schematic illustration of strategy used to generate *mylA*-complementary strain. Below pictures indicated PCR and qPCR analyses of C′ *mylA* strains.

Supplementary Table 1. ***Aspergillus*** **strains used in this study.**

Strain name	Relevant genotype	References
FGSC4	*A. nidulans* wild type, *veA*$^+$	FGSC[a]
RJMP1.59	*pyrG89; pyroA4; veA*$^+$	[1]
TNJ36	*pyrG89; AfupyrG*$^+$*; pyroA4; veA*$^+$	[2]
THS30	*pyrG89; AfupyrG*$^+$*; pyroA*$^+$*; veA*$^+$	[3]
TYE69.1~3	*pyrG89; pyroA4; ΔAN4618::AfupyrG*$^+$*; veA*$^+$	This study
THJ57.1~3	*pyrG89; pyroA4; ΔAN10944::AfupyrG*$^+$*; veA*$^+$	This study
TYE120.1~3	*pyrG89; pyroA4; ΔrgsA::AfupyrG*$^+$*; veA*$^+$	This study
TYE121.1~3	*pyrG89; pyroA4; ΔAN4618::AfupyrG*$^+$*; ΔrgsA::AnipyroA*$^+$*; veA*$^+$	This study
TYE72.1~2	*pyrG89; pyroA::mylA(p)::mylA::FLAG*$_{3x}$*::pyroA*[b]*; ΔmylA::AfupyrG*$^+$*; veA*$^+$	This study

[a] Fungal Genetic Stock Center

[b] The 3/4 *pyroA* marker causes targeted integration at the *pyroA* locus.

Supplementary References

1 Shaaban, M. I., Bok, J. W., Lauer, C. & Keller, N. P. Suppressor mutagenesis identifies a velvet complex remediator of *Aspergillus nidulans* secondary metabolism. *Eukaryot Cell* **9**, 1816-1824, doi:10.1128/EC.00189-10 (2010).

2 Kwon, N. J., Shin, K. S. & Yu, J. H. Characterization of the developmental regulator FlbE in *Aspergillus fumigatus* and *Aspergillus nidulans*. *Fungal Genet Biol* **47**, 981-993, doi:10.1016/j.fgb.2010.08.009 (2010).

3 Park, H. S. *et al.* Velvet-mediated repression of beta-glucan synthesis in *Aspergillus nidulans* spores. *Sci Rep* **5**, 10199, doi:10.1038/srep10199 (2015).
