# Supplementary material for: The MYB-like protein MylA contributes to conidiogenesis and conidial germination in Aspergillus nidulans
Source: Commun Biol. 2024 Jun 25;7:768. doi: 10.1038/s42003-024-05866-7 (PMC11199622; doi:10.1038/s42003-024-05866-7)
Supplement: Supplementary file 3 — Description of Additional Supplementary Files [file 42003_2024_5866_MOESM3_ESM.pdf]

Description of Additional Supplementary Files

File name: Supplementary Data 1

Description: List of differentially expressed genes between hyphae and conidia of *Aspergillus nidulans.*

File name: Supplementary Data 2

Description: List of differentially expressed genes in conidia of *mylA* deletion strain.

File name: Supplementary Data 3

Description: The source data behind the graphs in the paper.
